# Bridging the Gap: Modulatory Roles of the Grb2-Family Adaptor, Gads, in Cellular and Allergic Immune Responses

**DOI:** 10.3389/fimmu.2019.01704

**Published:** 2019-07-25

**Authors:** Deborah Yablonski

**Affiliations:** The Immune Cell Signaling Lab, Department of Immunology, Ruth and Bruce Rappaport Faculty of Medicine, Technion—Israel Institute of Technology, Haifa, Israel

**Keywords:** Gads, SLP-76, LAT, TCR, FcεRI, signal transduction, thymocyte development

## Abstract

Antigen receptor signaling pathways are organized by adaptor proteins. Three adaptors, LAT, Gads, and SLP-76, form a heterotrimeric complex that mediates signaling by the T cell antigen receptor (TCR) and by the mast cell high affinity receptor for IgE (FcεRI). In both pathways, antigen recognition triggers tyrosine phosphorylation of LAT and SLP-76. The recruitment of SLP-76 to phospho-LAT is bridged by Gads, a Grb2 family adaptor composed of two SH3 domains flanking a central SH2 domain and an unstructured linker region. The LAT-Gads-SLP-76 complex is further incorporated into larger microclusters that mediate antigen receptor signaling. Gads is positively regulated by dimerization, which promotes its cooperative binding to LAT. Negative regulation occurs via phosphorylation or caspase-mediated cleavage of the linker region of Gads. FcεRI-mediated mast cell activation is profoundly impaired in LAT- Gads- or SLP-76-deficient mice. Unexpectedly, the thymic developmental phenotype of Gads-deficient mice is much milder than the phenotype of LAT- or SLP-76-deficient mice. This distinction suggests that Gads is not absolutely required for TCR signaling, but may modulate its sensitivity, or regulate a particular branch of the TCR signaling pathway; indeed, the phenotypic similarity of Gads- and Itk-deficient mice suggests a functional connection between Gads and Itk. Additional Gads binding partners include costimulatory proteins such as CD28 and CD6, adaptors such as Shc, ubiquitin regulatory proteins such as USP8 and AMSH, and kinases such as HPK1 and BCR-ABL, but the functional implications of these interactions are not yet fully understood. No interacting proteins or function have been ascribed to the evolutionarily conserved N-terminal SH3 of Gads. Here we explore the biochemical and functional properties of Gads, and its role in regulating allergy, T cell development and T-cell mediated immunity.

## Introduction

### Gads—A Grb2-Family Adaptor Specialized for Immune Cell Signaling

**Figure 1:**
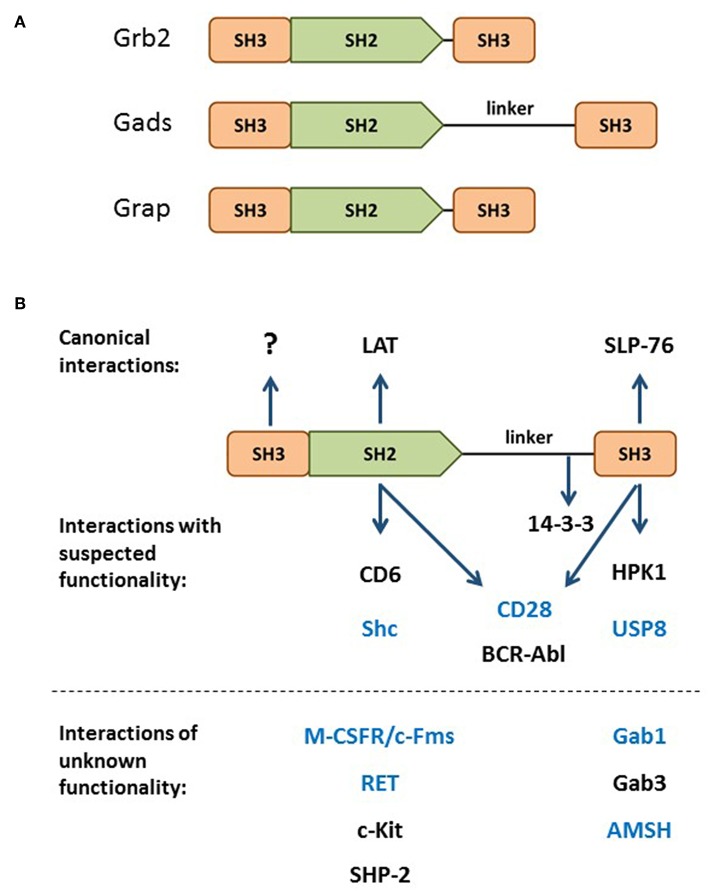
The Grb2 family. **(A)** Domain structure of the Grb2 family members: Grb2, Gads, and Grap. **(B)** Through its SH2 and C-terminal SH3, Gads bridges the antigen receptor-induced recruitment of SLP-76 to LAT (shown at top). Additional binding partners are listed below their relevant binding domain, with interactions of unknown functional relevance listed below the dotted line. The N-terminal SH3 has no known binding partner or regulatory function. Bait proteins that were used to identify Gads are shown in blue. 14-3-3 binds to a threonine phosphorylation site within the linker region. CD28 and BCR-Abl may interact with two Gads domains.

Gads is a hematopoietically-expressed adaptor protein that regulates T cell development, T cell-mediated immune responses and mast cell-mediated allergic responses. By virtue of its domain structure, Gads is a member of the Grb2 family of adaptors, which includes Grb2, Gads and Grap. This family is characterized by a central SH2 domain flanked by two SH3 domains, with Gads containing an additional glutamine- and proline-rich spacer between the SH2 and C-terminal SH3 domains (Figure 1A). Whereas Grb2 is ubiquitously expressed, Grap is primarily hematopoietic, and Gads is expressed exclusively in hematopoietic cell types, with particularly high expression in thymocytes, T cells, and mast cells, intermediate expression in monocytes, and no detectable expression in macrophages (1, 2). A low level of Gads expression has been detected in NK cells and in naïve murine and human B cells, where it is downregulated upon BCR stimulation (3, 4).

The signaling functions of Grb2-family adaptors are mediated by their conserved Src homology (SH) domains. SH2 is a structurally conserved modular domain, found in over 100 signaling proteins (5). Each SH2 domain contains a single binding cleft, which binds with moderate affinity to a characteristic phospho-tyrosine (pY) motif; in particular, Grb2-family SH2 domains bind to the pYxN motif (6).

The SH3 domain is another type of modular protein-protein interaction domain, found in hundreds of human proteins. SH3 domains typically bind with moderate affinity to canonical proline-rich motifs (7); however, the C-terminal SH3 of Gads binds with high affinity to a non-canonical RxxK motif (8–10).

In resting cells, Grb2 adaptors are found in the cytoplasm, where they bind constitutively to key signaling proteins via their SH3 domains. Grb2 utilizes both SH3 domains to bind to the Ras exchange factor, SOS (11). Gads does not bind to SOS (12, 13), but binds to a hematopoietic adaptor, SLP-76, via a high affinity interaction of its C-terminal SH3 with an RxxK motif in SLP-76 (9, 10, 14, 15).

#### One Adaptor With Many Names

Discovered in the late 1990s by six different groups, Gads was given six different names: Grb2-family adaptor downstream of Shc (Gads) (16), Grb2-related adaptor protein-2 (Grap2) (17), Monocyte Adaptor (Mona) (1), Grb2-related protein of the lymphoid system (GrpL) (12), Grb2 family member of 40 kD (Grf40) (18), and Grb2-related protein with insert domain (GRID) (19). Most of these groups cloned Gads by virtue of its ability to form stable protein-protein interactions via its SH2 or C-terminal SH3 domain. A number of groups identified Gads by screening cDNA expression libraries with pYxN-containing phosho-protein baits, such as Shc (16), Fms (1), RET (20), or the cytoplasmic tail of CD28 (19). In addition, Gads has been shown to bind via its SH2 to BCR-Abl and c-Kit (16), SHP-2 (12), and CD6 (21). Other groups identified Gads in the course of yeast two hybrid screens with bait proteins that contain an RxxK motif, such as Gab1 (17), AMSH (18), and, more recently, USP8 (also known as UBPY) (14, 22). In addition, the C-terminal SH3 of Gads can bind to RxxK motifs in HPK1 (23–25) and Gab3 (1). The known Gads-binding partners are summarized in Figure 1B.

#### The Bridging Function of Gads—Two Cell Types, One Signalosome

Soon after the identification of Gads, it became apparent that its main function is to serve as an antigen receptor-induced bridge between two other hematopoietic adaptors, LAT and SLP-76 (12, 13, 18) (Figure 1B). Gads performs this bridging function in two cell types: T cells and mast cells. These cell types have distinct developmental pathways, and use structurally distinct receptors to recognize different types of antigens; nevertheless, the signaling pathways triggered upon antigen recognition are remarkably similar.

**Figure 2:**
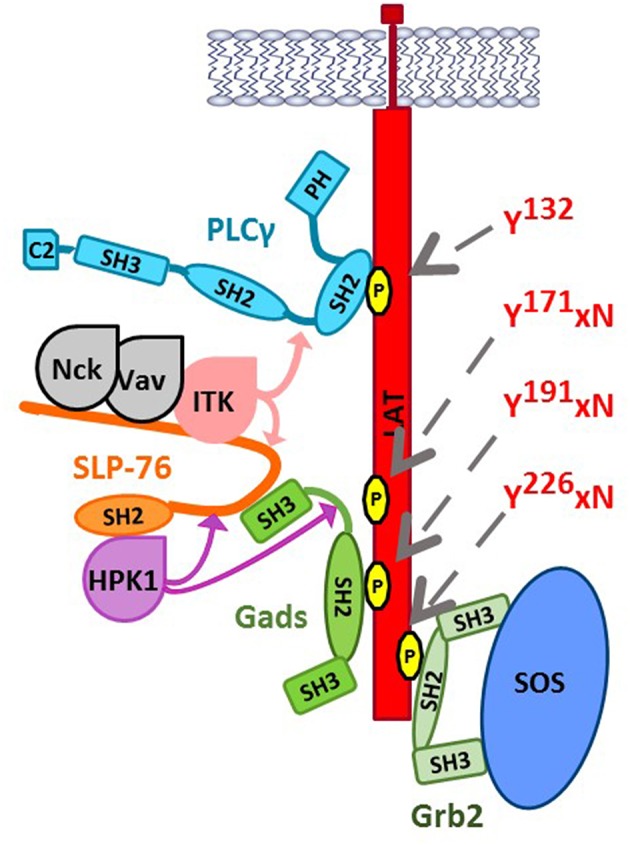
The LAT-nucleated signaling complex. Upon TCR stimulation, LAT is phosphorylated by ZAP-70 at Tyr^132^, Tyr^171^, Tyr^191^, and Tyr^226^ (indicated by gray arrows), triggering the SH2-mediated recruitment of key signaling proteins. PLC-γ1 binds directly to LAT at pTyr^132^. Grb2 bridges the recruitment of SOS to LAT. Gads binds cooperatively to pTyr^171^ and pTyr^191^, bridging the recruitment of SLP-76 and its associated binding partners to LAT. Nck, Vav, and Itk bind to N-terminal tyrosine phosphorylation sites on SLP-76 (Tyr^113^, Tyr^128^, and Tyr^145^), whereas HPK1 and ADAP (not shown) can bind to the C-terminal SH2 of SLP-76. Binding to SLP-76 is required for the catalytic activity of two SLP-76-associated kinases, Itk, and HPK1. Substrates of Itk within the signalosome are indicated by light pink arrows; Itk phosphorylates SLP-76 at Tyr^173^, which is required for subsequent Itk-mediated phosphorylation of PLCγ1 at Tyr^783^. HPK1 phosphorylates SLP-76 and Gads at negatively regulatory sites, indicated by purple arrows (SLP-76 Ser^376^ and Gads Thr^262^).

Recognition of antigen by the T cell antigen receptor (TCR) or the mast cell FcεRI triggers multi-site tyrosine phosphorylation of a trans-membrane adaptor, known as LAT, to which Grb2 and Gads bind via their SH2 domain. In this way, Grb2 recruits SOS to LAT, whereas Gads recruits SLP-76 to LAT. These events culminate in the antigen-induced assembly of a large, LAT-nucleated signaling complex, sometimes referred to as the LAT signalosome, which triggers downstream signaling events (Figure 2).

The superficially non-descript role of Gads as a bridge between two more famous adaptors, LAT and SLP-76 [reviewed in (26, 27)], may explain why Gads has not been reviewed in depth since 2001 (2), except in the context of the Grb2 family as a whole (28). Since this time, numerous studies have provided insight into the roles played by Gads and the regulatory inputs acting on Gads, which together warrant a closer look at this immune cell signaling molecule.

### Proximal Signaling by the T Cell Antigen Receptor—A Quick Overview

Each T cell expresses a clonotypic antigen receptor (TCR), specific for a particular combination of antigenic peptide and cell surface MHC molecule (pMHC). Within the cell, TCR ligation triggers a cascade of tyrosine kinases (29, 30), initiated by the Src-family kinase, Lck, which phosphorylates ITAM motifs within the TCR complex. Dual-phosphorylated ITAMs bind the Syk-family kinase, ZAP-70. Active ZAP-70 then phosphorylates LAT at least four sites, including Tyr^132^, Tyr^171^, Tyr^191^, and Tyr^226^ (31–34). Of these sites, PLC-γ1 binds directly to LAT pTyr^132^, whereas the three C-terminal sites conform to the Grb2 family-specific motif, pYxN (Figure 2).

In parallel, ZAP-70 phosphorylates SLP-76 at three N-terminal tyrosines (35–37), which bind to the adaptor, Nck, the guanine nucleotide exchange factor, Vav, and the Tec-family tyrosine kinase, Itk. Vav and Nck are responsible for TCR-induced changes to the actin cytoskeleton, whereas Itk participates in a signaling pathway leading to the activation of phospholipase C-γ1 (PLC-γ1). Finally, the C-terminal SH2 of SLP-76 binds to additional signaling proteins, including the adaptor ADAP (previously known as SLAP130/Fyb) and the serine threonine kinase HPK1. HPK1 phosphorylates two defined sites on SLP-76 and Gads (indicated by purple arrows in Figure 2), which will be discussed below.

One of the most important functions of the LAT signalosome is to mediate the TCR-induced activation of PLC-γ1. PLC-γ1 binds to LAT Tyr^132^, and is activated upon its phosphorylation on two tyrosine residues (38, 39). Itk-mediated phosphorylation of PLC-γ1 occurs by a sequential mechanism (indicated by pink arrows in Figure 2), in which Itk binds to and is activated by SLP-76 (40–42), then phosphorylates SLP-76 at Tyr^173^, a conserved site that is required for the subsequent Itk-mediated phosphorylation of PLC-γ1 (36, 43). Activated PLC-γ1 generates second messengers that trigger calcium increase and the activation of Ras and PKC, with consequent activation of calcium- and Ras-dependent transcription factors that are required for TCR-induced transcriptional changes (44).

## The Regulated Assembly and Disassembly of LAT-nucleated Signaling Complexes

### Cooperative Assembly of the LAT Signalosome

The LAT signalosome is a complex structure composed of many different proteins, which is rapidly assembled upon TCR stimulation. All four distal tyrosines of LAT are required for signalosome function (31–34), suggesting that a complete signalosome must be assembled. Biophysical measurements show that the three distal tyrosines can bind to Grb2 or Gads with comparable affinity (8); yet, in intact cells, at least two motifs are required for the stable recruitment of Grb2, and two particular motifs (Y171 and Y191) are specifically required for the stable recruitment of Gads into the signalosome (34). These observations suggest that signalosome assembly is promoted by cooperative binding events; however, the basis for cooperativity was not known.

#### Gads SH2-Mediated Dimerization Promotes Its Cooperative Binding to LAT

**Figure 3:**
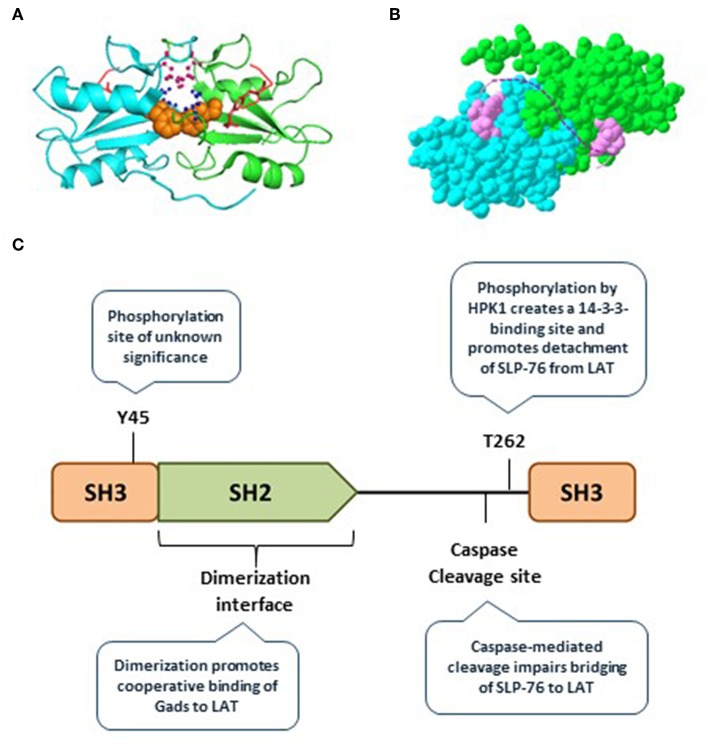
Gads regulatory sites include an SH2 dimerization interface. **(A)** The SH2 domain of murine Gads, cocrystalized with a short LAT peptide encompassing LAT pTyr^171^ [(6); PDB file 1R1P]. The minimal asymmetric unit includes two pairs of closely associated Gads SH2 domains, each bound to a pLAT peptide. Shown is the structure of one pair of SH2 domains (cyan and green chains), each bound to the LAT peptide (red). Three amino acid side chains from the putative dimerization interface are shown, including F92 (orange), R109 (pink), and D91 (blue). **(B)** A space-filling representation of the structure shown in **A**, revealing an extensive dimerization interface measuring ~850 Å^2^. The dotted pink line illustrates the possibility that the two bound pTyr peptides could represent dual binding sites on a single molecule of LAT, binding cooperatively to two molecules of Gads. **(C)** Summary of the currently known regulatory mechanisms converging on Gads, including Gads dimerization, HPK1-mediated phosphorylation of Gads, and caspase3-mediated cleavage of Gads. Tyr^45^ is a conserved TCR-inducible Gads phosphorylation site of unknown function, found within the N-terminal SH3 of Gads.

Recently, we discovered that the Gads protein undergoes spontaneous, reversible dimerization. Gads dimerization depends on its SH2 domain, and is further stabilized by additional domains found in full-length Gads (45). We used a structural model to identify the SH2 dimerization interface, and showed that it is distinct from the pTyr-binding pocket (45) (Figure 3A). This model suggests how paired binding of Gads to its dual binding sites on LAT may stabilize the dimeric configuration (Figure 3B). Consistent with this idea, competitive binding experiments revealed preferentially paired binding of Gads to a dual-phosphorylated LAT peptide, even in the presence of excess, competing single-phosphorylated LAT (45). Mutational inactivation of the dimerization interface reduced the preferentially paired binding of Gads to LAT, and impaired its ability to discriminate between single- and dual-phosphorylated LAT. In intact cells, disruption of the dimerization interface disrupted the antigen receptor-induced recruitment of Gads to phospho-LAT, moderately impaired TCR responsiveness in a model cell line, and profoundly impaired FcεRI-mediated activation of primary, bone marrow-derived mast cells (45).

#### Other Examples of Cooperativity at LAT

Assembly of the LAT signalosome appears to be driven by multiple cooperative binding events, including, but not limited to Gads dimerization. A recent study highlighted the cooperative assembly of LAT, Gads, SLP-76, and PLC-γ1 into a tetrameric complex centered around LAT Tyr^132^ and Tyr^171^ (46). Upon *in vitro* reconstitution of this binding complex, elimination of any one of the above components reduced the binding interactions between the other three. Further, cooperative interactions mediated by Grb2 are also likely to influence signalosome assembly. SH2-mediated dimerization of Grb2 can occur via a domain swapping mechanism, in which the C-terminal helix of the SH2 domain takes its place in a neighboring SH2 domain, thereby producing a stably intertwined dimeric form (47–49). It will be interesting to see whether Grb2 SH2 dimerization affects its binding to LAT, and how the competitive binding of Grb2 and Gads to overlapping sites on LAT eventually determines the overall structure and stoichiometry of the signalosome.

#### Why Are Cooperative Interactions at LAT so Important?

One insight may be seen in the recent observation that signaling through LFA-1 triggers phosphorylation of LAT at Tyr^171^ but not at Tyr^191^, Tyr^226^, or Tyr^132^. This selective phosphorylation allows LAT to bind to a Grb2-SKAP1 complex, but not to Gads-SLP-76 (50). The absence of binding to Gads-SLP-76 is consistent with the requirement for two sites to mediate the cooperative binding of LAT to Gads (34, 45). This observation further suggests that Gads cooperativity may allow cells to identify productive TCR activation, which leads to ZAP-70-dependent phosphorylation of LAT at four tyrosines. In contrast, initial scanning of the APC would lead to LFA-1-dependent phosphorylation of LAT at Tyr^171^ alone. It remains to be shown whether Tyr^171^ is in fact phosphorylated in the context of a transient, non-cognate interaction between a T cell and an APC.

### Signaling Microclusters Promote TCR Responsiveness

**Figure 4:**
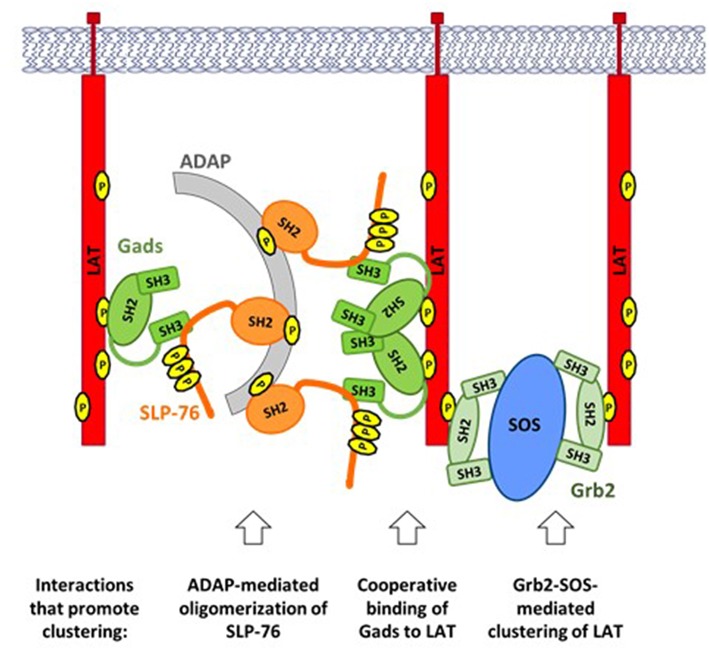
TCR-induced clustering of LAT. A web of multipoint, SH2-mediated interactions promotes the formation of microclusters, containing LAT, Gads, and SLP-76. ADAP-mediated oligomerization of SLP-76: Upon TCR stimulation ADAP is phosphorylated at three tyrosines that bind to the SH2 of SLP-76, leading to oligomerization of SLP-76 and its associated Gads. Cooperative binding of Gads to LAT: Gads SH2 dimerization promotes its cooperative binding to two adjacent sites on LAT, thereby recruiting ADAP-oligomerized SLP-76 to one or more LAT molecules. Grb2-SOS-mediated clustering of LAT: Each molecule of SOS can bind to two molecules of Grb2, each of which may bind to a different molecule of LAT, thereby bringing them into the growing cluster. Each of the above interactions can occur repeatedly, creating a web of interacting proteins that together stabilize a large signaling cluster.

Upon TCR stimulation, LAT-nucleated signaling complexes (Figure 2) are incorporated into larger (micrometer or sub-micrometer) structures, referred to as microclusters (51) (Figure 4). Microclusters containing SLP-76, LAT, and Gads appear rapidly at the site of TCR stimulation, followed by their microtubule-mediated translocation toward the center of the stimulatory contact site (52, 53). Live cell imaging revealed that the appearance of the first microclusters coincides with the initiation of calcium flux, suggesting that microcluster formation may be required for downstream signaling events (52).

The Grb2-SOS complex is an important driver of microcluster formation (Figure 4, right). Each SOS molecule can bind to two molecules of Grb2, which may bind to pTyr sites on adjacent molecules of LAT, thereby recruiting multiple molecules of LAT into the microcluster (11, 54). Since each LAT molecule can bind up to three molecules of Grb2, the potential for cluster formation by this mechanism is quite large.

In addition to Grb2 and SOS, both SLP-76 and Gads are required to support the formation, persistence and translocation of TCR-induced microclusters (53). The recruitment of SLP-76 into microclusters depends on two of its protein-interaction sites: its Gads-binding motif, and its C-terminal SH2 domain. Surprisingly, SLP-76 itself is required for the recruitment of Gads into microclusters (53). These results demonstrate that the Gads-mediated recruitment of SLP-76 to LAT is necessary, but not sufficient for microcluster formation; in addition, a ligand of the SLP-76 SH2 domain is required to recruit SLP-76 and its associated Gads into the clusters.

The SH2 domain of SLP-76 contributes to microcluster formation via its interaction with another adaptor protein known as ADAP (Figure 4, left). ADAP pre-localizes at TCR protrusions that may make contact with the APC (55). Upon TCR stimulation, ADAP is phosphorylated at three tyrosines that bind to SLP-76, resulting in the stable oligomerization of SLP-76 and its associated Gads (56). Oligomerized SLP-76 may then be recruited to LAT via the cooperatively paired binding of Gads to LAT pTyr^171^ and pTyr^191^ (45). In the context of ADAP-trimerized SLP-76, these Gads-mediated interactions may contribute to the cross-linking of two or more LAT molecules (Figure 4, center), similar to the cross-linking induced by Grb2-SOS. Consistent with this notion, all three ADAP tyrosines are required for the optimal assembly and stabilization of TCR-induced microclusters (56); whereas any microclusters that form in the absence of ADAP are immobile, smaller, and less persistent than those seen in wild-type cells (55, 57).

A common theme shared by all of the above mechanisms is that clustering is driven by the multipoint binding of individual signaling proteins to each other. Multipoint binding of Grb2 to SOS, Grb2 to LAT, SLP-76 to ADAP and Gads to LAT together create a web of interactions, linking individual signaling complexes into large microclusters that may amplify proximal signaling events to trigger profound downstream responsiveness.

### Negative Regulatory Pathways Intersecting at Gads

The multiple cooperative interactions occurring at LAT result in the formation of highly stable signaling complexes and microclusters (54). This cooperative network likely contributes to the sensitivity of TCR signaling and supports the binary (fully on or off) nature of T cell responses. Yet, the high stability of the physical signaling network suggests that dedicated regulatory pathways must be required to disassemble the complexes and microclusters in order to terminate signaling.

Microcluster disassembly appears to be a regulated, stepwise process. Gads and SLP-76 are among the first signaling molecules to exit the LAT-nucleated signaling microclusters (58), suggesting that disassembly may be initiated and regulated by pathways acting on Gads. Here we discuss two types of negative regulatory pathways that act on the linker region of Gads to promote signalosome disassembly and limit TCR responsiveness: HPK1-mediated phosphorylation of Gads, and caspase-mediated cleavage of Gads (Figure 3C).

#### HPK1 Mediates a Negative Feedback Loop, Activated by Gads and SLP-76

A member of the germinal center kinase (GCK) family, the serine/threonine kinase HPK1 (also known as MAP4K1) is expressed exclusively in hematopoietic cells and is activated upon TCR stimulation (59, 60). Lck, ZAP-70, LAT, and SLP-76 are all required for the TCR-induced activation of HPK1, suggesting that it forms part of the canonical TCR signaling pathway (60). Upon its activation, HPK1 appears to balance different downstream branches of the TCR response, by promoting the activation of JNK and NFκB, while simultaneously inhibiting the activation of Erk and AP1 (60–62).

Most importantly, HPK1 forms part of a negative feedback loop that limits signalosome activity. In the first part of the loop, SLP-76 acts within the LAT signalosome to mediate the activation of HPK1 (62). In the second part of the loop, HPK1 limits TCR responsiveness, by phosphorylating SLP-76 and Gads at negative regulatory sites, depicted by purple arrows in Figure 2 (63–66).

The TCR-induced activation of HPK1 occurs by a multistep mechanism, involving SLP-76 and Grb2-family adaptors. Upon TCR stimulation, a proline-rich region of HPK1, which can bind to Grb2-family adaptors, mediates its recruitment to LAT (24, 25, 59, 60). It is not yet clear exactly how HPK1 is recruited LAT; however, Gads binds weakly to HPK1 (23), suggesting that it is probably not the primary mechanism of recruitment. Once recruited to LAT, HPK1 is phosphorylated by ZAP-70 on Tyr^379^, which binds with high (~8 nM) affinity to the SH2 of SLP-76, and this interaction is essential for the catalytic activation of HPK1 (56, 62). Activation of HPK1 also depends on the Gads-binding motif of SLP-76 (62), further supporting the notion that HPK1 activation occurs within the LAT signalosome. Taken together, the evidence suggests that HPK1 activation depends on its Grb2-dependent recruitment to LAT, where it is phosphorylated by ZAP-70, and then binds to and is activated by the SH2 of SLP-76.

Active HPK1 exerts negative feedback by phosphorylating SLP-76 at Ser^376^ and Gads at Thr^262^ (63, 64). Both HPK1-targeted sites bind in a phosphorylation-dependent manner to 14-3-3 proteins, which appear to promote the detachment of SLP-76 and Gads from LAT microclusters (63, 64). In addition, phosphorylation of SLP-76 at S^376^ may trigger its ubiquitin-mediated degradation (67). Consistent with its negative regulatory function, HPK1-deficient primary T cells exhibit increased TCR-induced phosphorylation of SLP-76 and proliferation (66). Moreover, mutational inactivation of SLP-76 Ser^376^ or Gads T^262^ in a T cell line model resulted in increased TCR-induced phosphorylation of PLC-γ1, as well as increased TCR+CD28-induced transcription of the IL-2 gene (63, 65). Thus, HPK1 appears to activate a negative regulatory feedback pathway, which is at least partially mediated by phosphorylation of 14-3-3-binding sites on SLP-76 and Gads.

#### Caspase-Mediated Cleavage of Gads May Limit Immune Responsiveness

At later time points in the process of T cell activation, Gads activity is limited by caspase3-mediated cleavage, which occurs at a conserved DIND cleavage site found within the linker region of Gads (68, 69). Cleavage at this site separates the LAT-binding SH2 domain of Gads from the SLP-76-binding C-terminal SH3, and thus cleavage of Gads can strongly impair the recruitment of SLP-76 to LAT. In T cell lines, caspase3-mediated cleavage of Gads occurs approximately 1 h after stimulation of CD95, also known as the Fas receptor (68, 69). It is interesting to note that *in vivo* caspase3-mediated cleavage of Gads has been observed upon the induction of oral tolerance, and also upon treatment of primary T cells with anergizing stimuli; indeed the expression of a caspase3-resistant allele of Gads rendered T cells partially resistant to anergy (70, 71). These observations suggest that caspase-mediated cleavage of Gads may be part of the mechanism by which T cell tolerance is maintained.

## Evolutionary Conservation of Gads

Only two Gads domains, its central SH2 and C-terminal SH3, have been implicated in its role as a bridge between LAT and SLP-76. It is quite remarkable that Gads SH2 and C-SH3 have many potential binding partners (Figure 1B), whereas no interacting proteins or signaling functions have been ascribed to the N-terminal SH3 of Gads.

To gain insight into the relative importance of each Gads domain, we examined their evolutionary conservation, by aligning 66 vertebrate Gads orthologs (listed in Supplementary Table 1), representing a wide variety of taxonomical orders. A representative alignment of 14 Gads orthologs was color coded to indicate the extent of conservation among the 66 vertebrate species that we examined (Supplementary Figure 1). This analysis revealed remarkable conservation of all three SH domains of Gads. Fifty one percent of the N-SH3 residues, 60% of the SH2, and 48% of the C-SH3 residues were identical in over 95% of species examined (Supplementary Figure 1, yellow residues). When considering only 23 avian and 27 mammalian orthologs (excluding 3 marsupial orthologs), we found that 81% of N-SH3 residues, 77% of SH2 residues and 73% of C-SH3 residues were identical in over 92% of the species (Supplementary Figure 1, yellow and green residues). The remarkable evolutionary conservation of the N-terminal SH3 suggests that it performs an evolutionarily conserved, albeit currently unknown function, which may be mechanistically linked, or unrelated to the role of Gads as a bridge between SLP-76 and LAT.

The linker region varied between vertebrate classes, both in its sequence and in length. It was longest in mammalian orthologs (107–129 residues), due to the presence of a central proline-and glutamine-rich region of variable sequence, which was shorter or absent in the other vertebrate classes. The highest conservation within the linker sequence was observed among avian orthologs, in which it was of intermediate length (94–96 residues), and featured a central glutamine-rich motif, but no proline-rich motifs. No linker motifs were conserved among all vertebrate classes, but a motif encompassing the caspase cleavage site (RxGGSLDxxD) (68, 69) and another encompassing the threonine phosphorylation site (RRHTDP) (64) were conserved among mammalian and avian species (Supplementary Figure 1). Taken together these observations suggest that the linker region may serve a class-specific regulatory role that is not part of the core signaling function of Gads.

Overall, the high evolutionary conservation of Gads provides evidence for its important biological functions. To better understand its functions, it is instructive to compare the phenotypes of mice lacking Gads, to mice lacking LAT or SLP-76. As detailed below, the mast cell phenotypes of these mice are closely matched, consistent with the notion that the main role of Gads in mast cells is to serve as a bridge between LAT and SLP-76. In contrast, the T cell phenotypes are quite divergent, suggesting that Gads may play additional regulatory roles in T cells.

## Regulatory Roles of Gads *in vivo*

### The Mast Cell High-Affinity Receptor for IgE (FcεRI) Signals Through a LAT-Gads-SLP-76 Complex

Type I hypersensitivity is a common type of allergic response that occurs when sensitized mast cells respond to environmental antigens (also known as allergens) via their high affinity receptor for IgE (the FcεRI). Sensitization occurs upon the binding of allergen-specific IgE to the FcεRI, which thereby functions as an indirect antigen receptor. Subsequent exposure to allergen activates the FcεRI, triggering a cascade of events that is highly analogous to the TCR signaling pathway [reviewed in (72, 73)]. In brief, phosphorylated receptor ITAM motifs activate a Syk-family kinase that phosphorylates LAT and SLP-76, leading to the formation of a LAT-nucleated signalosome, bridged by Gads. These proximal events result in the activation of PLC-γ, with a consequent increase in intracellular calcium that triggers the release of preformed granules containing a variety of allergic mediators. Over a longer time scale, the FcεRI triggers the transcription, translation and release of inflammatory cytokines, including IL-6 and others.

Neither LAT, SLP-76, nor Gads are required for mast cell development. LAT-deficient mice had normal numbers of mast cells in the skin (74), SLP-76-deficient mice had normal numbers of mast cells in the skin and bronchi (75), and Gads-deficient mice had normal numbers of mast cells in the skin, stomach and peritoneal cavity (76). Mast cells can also be derived *ex vivo* by prolonged culture of bone marrow precursors in the presence of IL-3. Such bone marrow-derived mast cells (BMMCs) were readily obtained in the absence of LAT, SLP-76, or Gads (74–76), suggesting that all three adaptors are dispensable for mast cell differentiation and proliferation. Nevertheless, all three adaptors were required for FcεRI responsiveness, both *in vivo* and *ex vivo*.

To assess FcεRI responsiveness *in vivo*, endogenous mast cells are sensitized by treating mice with monoclonal IgE (anti-DNP), followed by intravenous administration of a DNPylated protein antigen. When assayed in this manner, SLP-76-deficient and LAT-deficient mice exhibited no sign of passive systemic anaphylaxis as measured by systemic release of histamine (74, 75). The response of SLP-76 deficient mice was limited to a mild and transient tachycardia whereas wild-type mice exhibited profound tachycardia that was lethal in 50% of the mice (75). Gads-deficient mice were similarly non-responsive when sensitized locally in the ear and assessed for passive cutaneous anaphylaxis in response to DNPylated antigen (76).

For in depth examination of FcεRI-induced signaling events, LAT-, SLP-76-, and Gads-deficient primary bone marrow-derived mast cell lines (BMMCs) were sensitized with monoclonal IgE (anti-DNP) and stimulated with a DNPylated protein antigen. FcεRI-induced phosphorylation of PLCγ was reduced in both LAT- and SLP-76-deficient BMMCs (74, 75), but to our knowledge was not assessed in Gads-deficient BMMCs. Downstream of PLCγ, calcium flux was markedly impaired in LAT-, SLP-76- and Gads-deficient BMMCs (74–76). Further downstream, the rapid FcεRI-induced release of preformed mediators was abrogated in SLP-76-deficient BMMC (75), and markedly decreased in LAT-deficient (74) and in Gads-deficient BMMC (76). The slower FcεRI-induced release of cytokines was virtually absent in SLP-76-deficient (75) and in Gads-deficient BMMC (76) and was markedly reduced in LAT-deficient BMMC (74).

Overall, the phenotypic similarity of LAT- Gads- and SLP-76-deficient BMMC suggests that the three adaptors function together to mediate FcεRI responsiveness. This conclusion is further supported by mutational analysis. Substitution of the four distal LAT tyrosines with phenylalanine (4YF) produced a mast cell phenotype equivalent to the loss of LAT (77), suggesting that FcεRI responsiveness depends on a LAT-nucleated signalosome. Further, the bridging function of Gads can be specifically ablated by a 20 amino acid deletion in SLP-76 (Δ224–244), which disrupts its interaction with Gads (13). This deletion precisely phenocopied a lack of SLP-76 in all mast cell assays (78–80), providing strong evidence that the BMMC-specific signaling functions of SLP-76 absolutely depend on its association with Gads. Finally, mutational inactivation of the Gads dimerization interface phenocopied a loss of Gads (45), suggesting that FcεRI responsiveness depends on the ability of Gads to bind cooperatively to the LAT signalosome. Taken together, these results strongly suggest that the most important signaling function of Gads in mast cells is to bridge the FcεRI-induced formation of the LAT signalosome, which is required for all downstream responses.

A possible caveat to the above conclusion relates to subtle differences in the phenotypes reported for LAT- Gads- and SLP-76-deficient BMMC. In particular, the impairment of FcεRI responsiveness appears to be most severe in SLP-76-deficient BMMC and somewhat milder in LAT-deficient BMMC. This difference may reflect the presence of LAT2, which can bind to Gads, and thereby partially compensate for the absence of LAT1 (81). Alternatively, the phenotypic differences may reflect subtle differences in experimental protocols, such as the strength of antigenic stimulation that was applied, and/or the method of data analysis. It is important to note that BMMC responses are often binary, such that the frequency of responding cells depends on the concentration of antigen applied, and strong stimulation can partially compensate for the lack of Gads (45). Similarly, SLP-76-deficient BMMCs exhibited barely detectable calcium flux when stimulated with a low dose of DNPylated antigen, whereas calcium flux was detectable in a small fraction of SLP-76-deficient cells upon stimulation with a 100-fold higher dose (75). It is therefore possible that the partial responsiveness of LAT-deficient BMMCs may reflect a binary response of a small population of cells upon stimulation with a relatively high concentration of antigen.

### The Essential Role of SLP-76 and LAT in the T Cell Lineage

#### A Quick Overview of Thymic Development

All T cell lineages develop in the thymus, from which a number of developmentally and functionally distinguishable subtypes emerge to the periphery. The two main subtypes are αβ and γδ T cells, which differ in the genetic loci that undergo rearrangement to produce the clonotypic TCR. αβ thymocytes cells further differentiate into the CD4 and CD8 lineages, including various subtypes of each. In all thymocyte lineages, signaling by the newly rearranged TCR drives thymocyte selection and maturation. Thus, mutations that impair TCR signaling necessarily impair thymocyte development.

**Figure 5:**
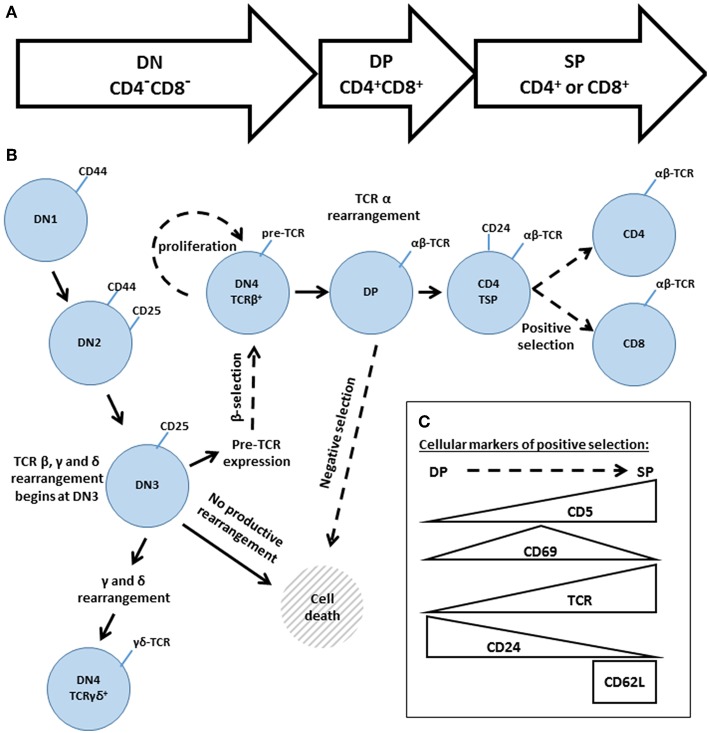
An overview of thymic development. **(A)** The three phases of thymocyte development, known as double negative (DN, CD4^−^CD8^−^) double positive (DP, CD4^+^CD8^+^) and single positive (SP, CD4^+^, or CD8^+^). **(B)** A higher resolution depiction, based on additional cell surface markers. Transitions regulated by Gads are indicated by dashed arrows. The DN phase can be subdivided into DN1 (CD44^hi^CD25^−^), DN2 (CD44^hi^CD25^+^), DN3 (CD44^lo^CD25^+^), and DN4 (CD44^lo^CD25^−^). TCR rearrangement begins during DN3. Successful rearrangement of TCRβ results in expression of the pre-TCR, composed of TCRβ, pre-Tα and CD3. At the β-selection checkpoint, pre-TCR signaling triggers cellular proliferation and transition through DN4 and on to the DP stage. DP cells rearrange TCRα, and the resulting mature αβ TCR may recognize self-pMHC ligands. High affinity recognition of pMHC triggers cell death through negative selection. Moderate affinity recognition triggers positive selection, accompanied by increased expression of CD5, CD69, and the TCR. Positively selected cells pass through a CD4 transitional single positive stage (TSP), characterized by high expression of CD24, and then transition into the mature SP compartment (CD4^+^ or CD8^+^ and TCR^hi^CD24^lo^CD62L^hi^). **(C)** Schematic summary of the markers of positive selection.

The development of conventional αβ T cells proceeds through three main stages: the double negative (DN: CD4^−^CD8^−^), double positive (DP: CD4^+^CD8^+^) and single positive (SP: CD4^+^ or CD8^+^) stages (Figure 5A). These broad phases can be further subdivided, based on additional cell surface markers (Figure 5B). DN thymocytes pass through four sub-stages (DN1-DN4, also known as proT1-proT4) that are distinguished by their expression of CD25 and CD44. As thymocytes enter DN3 (CD25^+^CD44^−^), they begin to rearrange the TCR β, γ and δ loci. Rearrangement of the γ and δ loci triggers progression to the γδ lineage; however, the vast majority of DN3 thymocytes rearrange the TCR β locus, resulting in expression of the pre-TCR, composed of the TCRβ, pre-Tα, and CD3 subunits. At the β-selection checkpoint, signaling pathways emanating from the pre-TCR trigger rapid thymocyte proliferation followed by transition through DN4 (CD25^−^CD44^−^) and on to the DP stage (82–84). DP thymocytes then rearrange the TCRα locus, resulting in the expression of a mature clonotypic αβ TCR. Recognition of self pMHC by the αβ TCR triggers intracellular signaling events that determine the cell fate (85–87). Moderate affinity interactions trigger positive selection, accompanied by changes in cell surface markers (Figure 5C), including increased expression of the TCR and passage to the SP compartment, whereas high affinity interactions trigger negative selection, leading to cell death and the removal of self-reactive T cell clones.

#### The Essential Role of LAT and SLP-76 in T Cell Development and Function

Consistent with their prominent role in TCR signaling, both LAT and SLP-76 are absolutely required for thymocyte maturation (88–90). Mice lacking LAT or SLP-76 are characterized by a small thymus, with cellularity reduced by 10-fold or more compared to wild-type. TCR β rearrangement proceeds normally, but the β-selection checkpoint is blocked due to impaired pre-TCR signal transduction, resulting in an accumulation of DN3 thymocytes, and a complete absence of all developmental stages beyond DN3. Consistent with these developmental defects, no mature αβ or γδ T cells can be detected in the periphery (88–90). Further supporting the severity of the signaling defect, treatment of LAT- or SLP-76-deficient mice with anti-CD3 did not trigger developmental progression beyond the DN stage (89, 90).

Conditional deletion of LAT or SLP-76 was used to characterize their role at later developmental stages. Deletion of either adaptor at the DP stage markedly impaired positive selection of thymocytes, as revealed by a profound block at the DP to SP transition. Further supporting this interpretation, LAT- or SLP-76-deficient thymocytes lacked the CD24^lo^TCRβ^hi^ and CD5^hi^ surface marker phenotypes, which characterize cells that have undergone positive selection. The impairment of positive selection was directly related to a lack of TCR signaling, as no calcium flux was observed upon TCR stimulation of SLP-76- or LAT-deficient DP thymocytes (91, 92). Similar results were observed upon conditional deletion of SLP-76 or LAT in mature, naïve T cells (93, 94). Absence of either adaptor abrogated short-term TCR responsiveness in all assays, including TCR-induced phosphorylation of PLC-γ and Erk, calcium flux, upregulation of activation markers, and TCR-induced proliferation, and also impaired homeostatic proliferation *in vivo* (93, 94). Consistent with these results, TCR signaling is profoundly blocked in LAT- and SLP-76-deficient T cell lines (95, 96). Taken together these phenotypes establish the necessity of SLP-76 and LAT for TCR responsiveness at all stages of T cell development, from earliest expression of the pre-TCR to the activation of peripheral T cells.

### The Puzzling Role of Gads in the T Cell Lineage

The severe phenotypes of LAT- or SLP-76-deficient mice (74, 75, 88–94), raise the question of whether these two adaptors function as part of an obligate signaling complex bridged by Gads, or whether they may exert some of their signaling functions independently of each other.

The answer to this question may depend on the cell type. As described above, Gads-deficient mast cells phenocopy LAT- or SLP-deficient mast cells (74–76); moreover, mutational analyses of the adaptors (45, 77–80) provide strong evidence that LAT, Gads and SLP-76 function as an obligate complex in the FcεRI signaling pathway.

The role of Gads in the T cell lineage is more complex. Gads is expressed in all T cells, beginning from the earliest stages of thymic development (97), yet T cell development and function appear to be partially independent of the bridging activity of Gads, as detailed below.

#### Mutational Studies Reveal TCR Signaling Pathways and Thymocyte Development Are Partially Independent of Gads

The partial dependence of TCR signaling on Gads was first noted upon stable reconstitution of a SLP-76-deficient, Jurkat-derived T cell line (J14) with wild-type or mutant alleles of SLP-76. Deletion of a 20 amino acid Gads-binding motif (Δ224–244) abrogated the binding of SLP-76 to Gads; nevertheless, TCR-induced tyrosine phosphorylation of SLP-76 was intact, and TCR responsiveness was markedly, but incompletely impaired (98). Complete impairment was observed upon substitution of the three N-terminal tyrosines of SLP-76 with phenylalanine (Y3F) (98). As another approach to reduce Gads-mediated signaling, overexpression of the Gads-binding motif (GBF) of SLP-76 competitively blocked the binding of Gads to SLP-76, resulting in impaired TCR-induced membrane recruitment of SLP-76, calcium flux, and upregulation of the CD69 activation marker (99). The role of Gads in mediating membrane recruitment of SLP-76 was directly addressed by fusing mutated SLP-76 to the transmembrane domain of LAT, thereby anchoring it to the membrane. Whereas signaling was disrupted by targeted point mutations that inactivate the Gads-binding motif of SLP-76, signaling was substantially rescued upon anchoring the mutated SLP-76 to the membrane, via its fusion to LAT (99).

Taken together, the T cell line-based experiments suggest that the interactions of SLP-76 with its N-terminal binding partners (Itk, Vav, and Nck) are absolutely required for TCR responsiveness. This SLP-76-nucleated complex appears to function best when recruited to LAT; nevertheless, SLP-76-dependent TCR signaling can proceed, albeit at reduced efficiency, in the absence of Gads-mediated recruitment. Consistent with this interpretation, upon deletion of Gads from the Jurkat T cell line, TCR-induced phosphorylation of SLP-76 remained intact, and TCR-induced PLC-γ phosphorylation, calcium flux and activation of NFAT were substantially reduced, but not eliminated (65). An important caveat to these conclusions is that cell line-based experiments involve strong stimulation of the TCR using an anti-TCR antibody, which may partially bypass the need for the bridging function of Gads.

Mouse models were used to explore the importance of Gads-mediated signalosome assembly under the strength of TCR stimulation that naturally occurs during thymic development. *In vivo* signalosome assembly can be disrupted in many different ways: by mutating the Gads-binding sites on LAT, by deletion or competitive inhibition of the Gads-binding site on SLP-76, or by deletion of Gads itself. Since LAT and SLP-76 are absolutely required for passage through the β-selection checkpoint, a reasonable estimation of TCR signaling competence can be based on the ability of thymocytes to develop beyond the DN3 stage.

LAT contains 10 conserved tyrosine residues, of which the four distal tyrosines (labeled in Figure 2) appear to be necessary and sufficient for signalosome assembly (31–34). Substitution of all four with phenylalanine phenocopied the absence of LAT in thymic development (100), as did substitution of only the three most-distal tyrosines (34, 101). In retroviral reconstitution experiments, a LAT construct containing only the four distal tyrosines supported TCR-induced binding of LAT to Gads, Grb2 and PLC-γ, and supported thymic development *in vivo* (34). Surprisingly, upon mutation of either of the Gads binding sites (Y171 or Y191), binding to Gads was lost, but thymic development was largely intact, resulting in the production of DP and SP thymocytes as well as peripheral CD4 and CD8 T cells (34). Given the inherent variability of retroviral reconstitution, it is impossible to estimate the degree to which this LAT mutant phenocopies wild-type LAT; however, this experiment certainly suggests that the LAT-Gads interaction is not absolutely required for thymic development.

To more precisely address the contribution of Gads bridging activity to T cell development and function, SLP-76-deficient mice were stably reconstituted with wild type or mutant alleles of SLP-76 (102, 103). Whereas wild-type SLP-76 fully supported thymic development, deletion of the Gads binding site (Δ224–244) resulted in an incomplete block at the DN3 to DN4 transition with ~75% reduction in thymic cellularity; nevertheless, DP and SP thymocytes were present, as well as peripheral CD4 and CD8 T cells (102, 103). In addition to affecting T cell development, the Δ224–244 mutation markedly impaired TCR-induced phosphorylation of PLC-γ1 (103) but only moderately reduced consequent downstream responses, including TCR-induced calcium flux, expression of the CD69 and CD5 activation markers, and proliferation (102, 103).

The above mutational analyses demonstrate that the TCR signaling functions of SLP-76 and LAT are only partially dependent on the bridging activity of Gads. Multiple lines of genetic evidence demonstrate that assembly of the LAT-Gads-SLP-76 signalosome promotes TCR responsiveness, but is not absolutely required for TCR signaling nor for thymic development.

#### Modulatory Role of Gads in T Cell Development and Function

Consistent with the above mutational analyses, Gads-deficient mice exhibit substantial, but incomplete defects in thymic development. Whereas LAT- or SLP-76-deficient mice arrest thymic development the DN3 to DN4 transition (88–90), Gads-deficient mice exhibit a partial block at this transition (97, 104), as well as defects in positive and negative selection (105, 106). Overall, thymic cellularity is reduced by 4-fold or more, including marked reductions in the total number of DP and SP thymocytes and a reduced ratio of CD4 to CD8 SP cells in the thymus and in the periphery (97, 106). Thus, Gads regulates multiple stages of thymic development, indicated by dashed arrows in Figure 5B, and detailed below.

##### Role of Gads at the β-selection checkpoint

The earliest steps of thymic development appear to be largely independent of Gads. As thymocytes enter DN3, they begin to rearrange the TCR β, γ, and δ genes (107, 108). Gads-deficient mice show no reduction in the absolute number of TCRβ^+^ or TCRγδ^+^ DN3 thymocytes, demonstrating that Gads is not required for TCR gene rearrangement (104). Subsequently, TCRβ^+^ and TCRγδ^+^ DN3 thymocytes differ markedly in their requirement for Gads at the DN3 to DN4 transition. Within the αβ lineage, the β-selection checkpoint is markedly impaired in the absence of Gads, resulting in a ~20-fold reduction in the number of TCRβ^+^ DN4 thymocytes compared to wild-type mice (104). In contrast, Gads-deficient TCRγδ^+^ DN3 thymocytes progress efficiently to DN4, and the number of TCRγδ^+^ DN4 thymocytes is reduced by <2-fold compared to wild-type mice (104). The incomplete developmental block of Gads-deficient mice at the DN3 to DN4 transition may therefore be partly explained by Gads-independent progression of TCRγδ^+^ thymocytes, which ultimately constitute 50% of the DN4 compartment in Gads-deficient mice, but only 10% in wild type mice (104).

The β-selection checkpoint depends on signals emanating from the pre-TCR, which trigger rapid proliferation and passage through DN4 and into the DP compartment (83). This developmental transition can be accelerated by *in vivo* administration of anti-CD3, which induced the advancement of virtually all wild-type DN3 thymocytes into the DN4 compartment; in contrast, <5% of Gads-deficient DN3 thymocytes advanced to DN4 upon anti-CD3 treatment (106). To better assess pre-TCR responsiveness under physiologic levels of signaling, Zeng et al. (104) measured cell cycling in TCRβ^+^ DN thymocytes. Upon the appearance of TCRβ, wild-type and Gads-deficient DN3 thymocytes exhibited a comparable increase in cell cycling, possibly indicating some degree of Gads-independent pre-TCR responsiveness. Subsequently, as thymocytes reduce expression of CD25 and transit to the DN4 compartment, wild-type cells continued cycling, whereas Gads-deficient cells exhibited decreased cycling and increased apoptosis. Taken together, the results suggest that Gads is required for sustained pre-TCR-induced proliferation and/or survival upon transition to DN4.

##### Role of Gads in positive and negative selection

Despite the marked impairment of β-selection, roughly three quarters of Gads-deficient thymocytes can be found in the DP compartment (97, 106). This unexpected observation suggests a bottleneck. Cells may be slow to enter the DP compartment due to impaired β-selection, but are also slow to exit this compartment, due to impaired positive and negative selection (Figure 5B, dashed arrows).

Positive and negative selection depend on signals emanating from the mature αβ TCR as it recognizes self pMHC ligands (109, 110). The TCR responsiveness of Gads-deficient thymocytes was examined upon *ex vivo* stimulation with anti-CD3. Whereas CD3-induced phosphorylation of SLP-76 was largely intact, the phosphorylation of PLC-γ and subsequent calcium flux were undetectable (106). When administered *in vivo*, anti-CD3 can mimic negative selection by persistently activating the TCR, triggering the deletion of DP thymocytes; however, no such deletion was observed in Gads-deficient mice (106). Mice carrying the HY TCR transgene, specific for the male-expressed H-Y antigen, are commonly used to assess positive and negative selection in a more physiologic setting. Whereas HY^+^ thymocytes are deleted by negative selection in wild-type male mice, they were not deleted in Gads-deficient male mice; moreover, positive selection was profoundly impaired in HY^+^ female mice (106). Together, the results suggest that in the absence of Gads, positive and negative selection are profoundly impaired due to impaired SLP-76-mediated signaling.

Closer examination of positive selection revealed that Gads-deficient DP thymocytes can advance as far as the CD4^+^ transitional SP (TSP) compartment (CD4^+^CD8^−^CD24^hi^) (105). The vast majority of Gads deficient TSP thymocytes retain an unactivated phenotype (TCRβ^lo^CD69^lo^), suggesting that have not yet undergone positive selection (105). Further advancement to the mature (CD24^lo^TCRβ^hi^CD62L^hi^) CD4 SP compartment is profoundly impaired, whereas advancement to the mature CD8 compartment is only moderately impaired (105). The defect in positive selection was confirmed using additional transgenic TCR models, including the MHC type II-restricted OT-II model and the MHC type I-restricted OT-I and P14 transgenic TCR models. Gads-deficient OT-II thymocytes arrested in the CD4 TSP compartment, reflecting a severe defect in positive selection (105). In contrast, Gads-deficient OT-I and P14 thymocytes were profoundly, but incomplete arrested in the CD4 TSP compartment, with the development of mature CD8 SP thymocytes and peripheral naïve CD8 T cells bearing the transgenic TCR (105). Taken together the results suggest that MHC class II-mediated positive selection is strongly dependent on Gads, possibly explaining the reduced ratio of CD4 to CD8 SP thymocytes observed in Gads-deficient mice.

##### Surprising peripheral T cell phenotypes of Gads-deficient mice

Consistent with their thymic defects, Gads-deficient mice have reduced numbers of peripheral CD4 and CD8 T cells, which exhibit moderately reduced expression of the TCR (97, 106). The ratio of peripheral CD4 to CD8 T cells is markedly reduced, reflecting the reduced production of mature CD4 SP cells in the thymus. Moreover, Gads-deficient peripheral CD4 T cells exhibit increased homeostatic proliferation accompanied by increased cell death, which together result in their reduced persistence in the periphery (97). This defect was not observed in peripheral CD8 T cells, which gradually increase in number as the mice age (97).

It is important to note that most of the peripheral T cells found in Gads-deficient mice have a non-naïve phenotype. Gads-deficient CD4 T cells tend to have an activated phenotype (CD44^hi^CD62L^lo^CD69^hi^), whereas virtually all of the CD8 T cells have a memory phenotype (CD44^hi^CD62L^hi^CD69^lo^) (97). Consistent with their non-nave phenotype, Gads-deficient CD4 and CD8 T cells exhibited an enhanced ability to produce IFNγ upon *ex vivo* stimulation with PMA and ionomycin (97). These observations clearly suggest that Gads-deficient peripheral T cells represent an altered developmental pathway, and may not be directly comparable in their subset composition or function to wild-type peripheral T cells.

Despite the multiple developmental defects within the T cell lineage, Gads-deficient peripheral T cells appear to be capable of supporting a degree of immune responsiveness. Seconds after TCR stimulation, TCR-induced calcium flux was profoundly impaired in Gads deficient CD4 and CD8 T cells; nevertheless a clearly detectable minority of cells responded when the concentration of stimulant was increased (97). Consistent with this observation, Gads-deficient OT-I T cells proliferated upon stimulation with their cognate peptide antigen (SIINFEKL); however, the threshold concentration of antigen required to induce multiple rounds of proliferation was increased by ~100-fold (111). Most surprisingly, Gads-deficient mice respond to immunization, producing antigen-specific IgM and IgG at levels comparable to wild-type mice (76). This result suggests that T cell helper activity may remain intact in the absence of Gads; however, it is not yet clear whether the helper activity is provided by conventional or innate-like T cell subsets. Taken together, it appears that Gads-deficient T cells can support immune responsiveness, provided that the dose of stimulating antigen is sufficiently high.

## Can we solve the puzzle of Gads?

The complex thymic and peripheral T cell phenotypes observed in Gads deficient mice are substantially milder than the arrested thymic development of LAT- and SLP-76 deficient mice. Whereas SLP-76 and LAT-deficient-mice completely lack peripheral T cells, Gads-deficient mice retain peripheral T cells that are capable of mediating a degree of immune responsiveness. Given that Gads serves as a TCR-inducible bridge between SLP-76 and LAT, how can we explain this marked divergence of phenotypes?

Here we shall discuss evidence supporting four possible explanations, which are not mutually exclusive. (1) Partially redundant mechanisms may allow for Gads-independent recruitment of SLP-76 to LAT; (2) Certain signaling functions of LAT and SLP-76 may be independent of their association; (3) A Gads-dependent branch of the TCR signaling pathway may selectively regulate the development of particular T cell subsets; and (4) Gads may perform regulatory functions outside of the LAT signalosome.

### Explanation #1: Partially Redundant Mechanisms May Allow for Gads- Independent Recruitment of SLP-76 to LAT

Without a doubt, Gads provides the most efficient and sensitive mechanism for recruiting SLP-76 to LAT, due to the high-affinity, constitutive binding of SLP-76 to Gads (18), coupled with the cooperatively stabilized binding of Gads to phospho-LAT (45). Nevertheless, a weak, TCR-inducible interaction of SLP-76 with phospho-LAT can be detected in the absence of Gads (65, 112). This barely detectable interaction suggests that compensatory mechanisms for recruiting SLP-76 to LAT may account for the residual TCR signaling activity observed in Gads-deficient cells.

#### Can Other Grb2 Family Members Compensate for the Lack of Gads?

One possible compensatory mechanism is the Gads-independent recruitment of SLP-76 to LAT by other Grb2-family members, Grb2 or Grap. Both Gads and Grb2 can bind via their C-terminal SH3 domains to a non-canonical RxxK motif found in SLP-76; however, Gads binds this motif with high affinity, measured at 9-20 nM (8, 10, 46), whereas Grb2 binds with ~500-fold lower affinity (10, 14, 15). Due to its low affinity, Grb2 cannot interact with SLP-76 in the presence of competing Gads (18); however, the absence of Gads may permit weak, Grb2-mediated recruitment of SLP-76 to LAT. A third family member, Grap, co-purifies with both SLP-76 and LAT (113); albeit the affinity of these interactions have not been reported. Any such compensatory recruitment may be cooperatively stabilized by additional interactions occurring within the LAT-nucleated signalosome. For example, binding of the SH3 domain of LAT-bound PLC-γ1 to a proline-rich motif in SLP-76 can substantially stabilize the recruitment of SLP-76 to LAT (46, 98). While consistent with previously reported protein-protein interactions, neither Grb2- nor Grap-mediated recruitment of SLP-76 to LAT has been directly demonstrated.

#### Can SH2-Mediated Clustering of SLP-76 Compensate for the Lack of Gads?

The Grb2-based mechanism of recruitment cannot explain the substantial signaling activity exhibited by SLP-76Δ224-244 (98, 102, 103), a mutant allele that lacks the RxxK motif, and therefore cannot bind to Gads or to Grb2. SLP-76Δ224-244 supports a degree of thymic development that is roughly comparable to mice lacking the SH2 domain of SLP-76 (102, 103). One possible interpretation of these results is that the SH2 domain and RxxK motifs of SLP-76 may mediate partially redundant mechanisms for recruiting SLP-76 to LAT.

ADAP is an adaptor that mediates clustering of SLP-76 via its multipoint binding to the SH2 domain of SLP-76 (Figure 4). We suggest that ADAP-mediated oligomerization of SLP-76 may partially compensate for the absence of Gads. We further note that ADAP binds to SKAP1, which may be recruited to LAT via its interaction with Grb2 (50). This speculative chain of interactions (SLP-76-ADAP-SKAP1-Grb2-LAT) is built on known protein-protein interactions, but its ability to recruit SLP-76 to LAT has not been directly demonstrated.

### Explanation #2: Certain Signaling Functions of LAT and SLP-76 May Be Independent of Their Association

While intriguing, the above-described compensatory mechanisms are not sufficient to explain the puzzle of Gads. The proposed mechanisms are quite weak, as the TCR-induced recruitment of SLP-76 to LAT is barely detectable in the absence of Gads. How then can we explain the relatively mild thymic phenotypes of Gads-deficient mice?

We suggest that separate SLP-76- and LAT-nucleated signaling complexes can form in the absence of Gads, and may mediate a degree of TCR responsiveness in the absence of their stable association. Indeed, the TCR-induced phosphorylation of SLP-76 and LAT are intact in Gads-deficient thymocytes (106) and T cell lines (65, 112). Further, some SLP-76 dependent signaling events are intact in Gads-deficient T cell line. Gads was not required for TCR-induced activation of AKT, nor was it required for TCR-induced actin polymerization or adhesion to the APC (112). SLP-76-dependent activation of Itk was likewise intact in Gads-deficient T cells, as evidenced by the intact TCR-induced phosphorylation of SLP-76 at its Itk-targeted site, Tyr^173^ (36, 40, 65).

Nevertheless, certain functions of LAT and SLP-76 are clearly dependent on Gads. Most importantly, Itk-mediated phosphorylation of PLC-γ1 was markedly reduced, and consequent downstream responses, including TCR-induced calcium flux, activation of the NFAT transcription factor, secretion of IL-2 and expression of the CD69 activation marker were substantially reduced in the absence of Gads (65).

These results provide evidence that one of the most important biochemical functions of Gads is to facilitate the interaction of SLP-76-bound Itk with its substrate, LAT-bound PLC-γ1 (40, 65). Gads accomplishes this by recruiting SLP-76 to LAT, thereby bringing Itk into close proximity with its substrate. Under conditions of high-intensity TCR stimulation, large numbers of SLP-76- and LAT-nucleated signaling complexes may accumulate and interact transiently via random diffusion, thereby bypassing the need for Gads. Consistent with this notion, Gads appears to be most important under conditions of low-intensity TCR stimulation, where Gads expression increases the frequency of responding cells (65, 111).

### Explanation #3: A Gads-Dependent Branch of the TCR Signaling Pathway May Selectively Regulate the Development of Particular T Cell Subsets

Any functional description of Gads must account for the non-naïve, CD44^hi^ phenotype exhibited by Gads-deficient peripheral T cells (97). This phenotype presents something of a paradox, as it suggests that the cells are antigen-experienced, and therefore TCR responsive; yet Gads-deficient thymocytes and peripheral T cells exhibit profoundly impaired TCR responsiveness (97, 106).

A similar non-naïve peripheral phenotype has been observed in mice lacking the Tec-family kinase Itk (114–116), and also in mice bearing a mutation at SLP-76 Tyr^145^ (117), a site that is implicated in the binding and activation of Itk. As described above, one of the most important biochemical functions of Gads is to facilitate the interaction of SLP-76-bound Itk with its substrate, LAT-bound PLC-γ1 (40, 65). Studies of Itk-deficient mice suggest a number of ways in which reduced signaling through the Gads-SLP-76-Itk branch of the TCR signaling pathway may result in the accumulation of non-naïve peripheral T cells.

#### Reduced Signaling Through Gads-SLP-76-Itk May Result in a Skewed TCR Repertoire

One potential source of a non-naïve phenotype may be the presence of self-reactive T cell clones in the periphery. Whereas self-reactive thymocytes are normally eliminated by negative selection, partial impairment of TCR signaling in the DP compartment may shift the boundary between positive and negative selection (109, 118), thereby allowing the maturation of self-reactive SP thymocytes.

This type of repertoire shift was observed in mice lacking the Tec-family tyrosine kinases, Itk and Rlk, and expressing the HY transgenic TCR (119). Whereas HY^+^ TCR-transgenic thymocytes are negatively selected by their cognate antigen in male wild-type mice, they were positively selected in Itk^−/−^Rlk^−/−^ male mice, suggesting that partially impaired TCR signaling resulted in the conversion of a negatively-selecting self-antigen into a positively-selecting TCR ligand (119). An analogous shift in the selection boundary may occur in Gads-deficient thymocytes; however, this has not been directly demonstrated.

Once in the periphery, constitutive exposure of self-reactive Gads-deficient T cells to their cognate self-antigen may result in a non-naïve phenotype. Consistent with this explanation, OT-I mice, in which the TCR repertoire is fixed, produce peripheral CD8 T cells with a naïve phenotype, even in mice lacking Itk or Gads (105, 114). Taken together, these results suggest that impairment of the Gads-SLP-76-Itk branch of the TCR signaling pathway results in a non-naïve phenotype that is at least partially due to an altered TCR repertoire.

#### Reduced Signaling Through Gads-SLP-76-Itk May Skew Additional Thymic Developmental Decisions

In addition to influencing the TCR repertoire, the impaired positive selection observed in Gads- or Itk-deficient mice may impede the development of conventional αβ T cells, while favoring the development of specialized T cell sub-populations with innate-like phenotypes. Mice lacking Itk exhibit altered thymic selection which results in the maturation of CD4 and CD8 T cells with an innate-like phenotype (114–116, 120, 121). Innate-like T cells are characterized by the constitutive expression of activation markers such as CD44 and may bear additional markers including CD122, NK1.1, and others. The peripheral T cells found in Gads-deficient mice bear certain characteristics of innate-like T cells, including relatively low expression of the TCR, an activated phenotype found on most Gads-deficient CD4 T cells (CD44^hi^CD62L^lo^CD69^hi^), a memory phenotype exhibited by virtually all Gads-deficient CD8 T cells (CD44^hi^CD62L^hi^CD69^lo^), and an enhanced ability to produce IFNγ upon *ex vivo* stimulation with PMA and ionomycin (97). Further characterization of additional cell surface markers, transcription factors and functional responses that characterize different innate-like T cell subsets (122) will be required to determine whether the peripheral T cells found in Gads-deficient mice are in fact innate-like, or merely activated conventional T cells. Finally, an inducible deletion of Gads will be required to determine the point of origin of the innate-like phenotype, which may be generated by altered selection in the thymus, or by increased homeostatic proliferation in the periphery.

### Explanation #4: Gads May Perform Additional Regulatory Functions Outside of the LAT Signalosome

Until now, we have viewed Gads exclusively as a component of the LAT-nucleated signalosome. Here, we consider the possibility that Gads may perform additional regulatory functions via its SH2-mediated interactions with other types of signaling complexes. The SH2 of Gads is specific for pYxN motifs, which are found in a variety of signaling proteins. Whereas many molecules of Gads are recruited to LAT, others may bind to costimulatory receptors such as CD28 and CD6, or to cytoplasmic signaling complexes nucleated by Shc (Figure 1B). Each of these Gads-binding partners plays a role in modulating T cell development and activation, and it is possible that some of their activity is mediated through Gads. According to this notion, the complex phenotype of Gads-deficient mice is the result of partial impairment of LAT-mediated signaling, combined with the impairment of additional Gads-dependent signaling pathways.

#### Does CD28 Costimulatory Activity Depend on Gads?

It has been known for some time that Gads can bind to a membrane-proximal pYMNM motif found in the cytoplasmic tail of the T cell costimulatory receptor, CD28 (19). The functional significance of this interaction is not clearly established, as the same pYMNM motif binds to PI3K and to Grb2 (123). To our knowledge, no study has addressed the CD28 signaling competence of a Gads-deficient T cell line or mouse, and so the contribution of Gads to CD28 signaling remains speculative.

The evidence implicating Gads in CD28 signaling is mixed. The cytoplasmic tail of CD28 includes two PxxP motifs, which are required to support the binding of CD28 to Gads in intact cells, and are likewise required for CD28 activity in some assay systems (124). This mutational evidence suggested an involvement of Gads in mediating CD28 costimulation; however, a subsequent binding study failed to confirm a direct dependence of Gads binding on the PxxP motifs (125). Indeed, a more recent study suggests that CD28 signaling depends specifically on its interaction with Grb2 (126). Based on current knowledge its seems unlikely that any aspect of the Gads-deficient phenotype can be directly attributed to reduced signaling through CD28.

#### What Is the Role of Gads in CD6 Costimulation?

CD6 is a T cell surface glycoprotein that binds to CD166 on antigen presenting cells. CD6 appears to combine costimulatory and inhibitory activities, which together may set the threshold for T cell activation (127, 128). Upon TCR stimulation, the long cytoplasmic tail of CD6 is tyrosine phosphorylated at multiple sites, two of which bind to the SH2 domains of SLP-76 and Gads (21, 113, 127). Direct binding studies confirmed that CD6 Tyr^662^ binds to the SH2 of SLP-76, whereas Tyr^629^ binds to the SH2 of Gads (21). Within intact cells, the high affinity constitutive association of SLP-76 and Gads promotes their cooperative, bivalent binding to CD6. Thus, the binding of Gads to CD6 depends on SLP-76, whereas mutation of CD6 at either Tyr^662^ or Tyr^629^ abrogates its binding to both SLP-76 and Gads (21).

The costimulatory activity of CD6 is revealed by experiments in which co-ligation of CD6 with CD3 augments TCR-induced production of IL-2. This costimulatory activity depends on the SLP-76 binding site, Tyr^662^ and the Gads binding site, Tyr^629^ (21). To further test the importance of these interactions, a C-terminal fragment of CD6 encompassing the binding sites for SLP-76 and Gads was fused to a CD19-specific chimeric antigen receptor (CD19-CAR). Inclusion of the CD6 fragment in the CAR construct increased CAR-induced production of IFN-γ as well as CAR-mediated killing of CD19-expressing cells (129). While intriguing, the role of SLP-76 and Gads in mediating increased potency of the CAR construct remains to be determined. More importantly, to date, no study has assessed CD6-mediated costimulatory activity in Gads-deficient mice or T cells; therefore, the possible contribution of Gads to CD6-mediated signaling remains to be definitively demonstrated.

#### What About Gads Binding to Shc?

Over 20 years ago, Shc-derived pTyr peptide baits were used to clone Gads from a cDNA expression library; indeed, the name of Gads (Grb2-family adaptor downstream of Shc) denotes its SH2-mediated binding to Shc (16). Soon thereafter, it became apparent that Gads SH2 domain binds to LAT (13, 18), and the implications of its interaction with Shc were not further explored. Given the marked phenotypic divergence between LAT- and Gads-deficient mice, it may be worthwhile to re-explore the mechanistic connections between Gads and Shc.

The Shc family of adaptor proteins has three members, but only ShcA is expressed in T cells, where it has two isoforms, p45 and p52. ShcA is a ubiquitously expressed protein, characterized by an N-terminal PTB domain, a C-terminal SH2 domain and a central region containing three tyrosine phosphorylation sites, two of which can bind to Grb2-family adaptors (130). ShcA is required for fetal development past embryonic day 11.5 (131); however, it may also have specific functions in the T cell lineage.

Some evidence implicates ShcA in the activation of T cell lines, as both isoforms are rapidly tyrosine-phosphorylated in response to TCR stimulation (132) and in response to incubation with IL-2 (133). Moreover, a Shc-deficient Jurkat-derived T cell line exhibits impaired TCR-induced activation of C-Rel, resulting in impaired TCR-induced production of IL-2 (134).

The specific role of ShcA in T cell development was studied by T-cell specific impairment of its function *in vivo*. In one approach, a mutant ShcA transgene lacking three tyrosine phosphorylation sites (ShcFFF) was conditionally expressed in the T cell lineage (135). ShcFFF may serve as a dominant negative allele, as it can interact with specific binding partners via its PTB and SH2 domains, but cannot recruit Grb2-family adaptors into the signaling complex. When expressed from the earliest stages of thymocyte development, the ShcFFF transgene essentially phenocopied Gads-deficient mice. Like-Gads-deficient mice, ShcFFF-expressing mice have a thymus that is 3 to 10-fold smaller than wild-type due to a partial block at the DN3 to DN4 (β-selection) checkpoint; further, this block is associated with reduced proliferation of DN3 and DN4 cells, and cannot be overcome by the *in vivo* administration of anti-CD3 (97, 104, 106, 135). A similar phenotype was observed upon conditional deletion of Shc in the DN compartment (135). Based on these results, we must consider the possibility that the phenotype of Gads-deficient mice is at least partially due to reduced signaling through Shc.

## Future Directions

Further research will be required to solve the puzzle of Gads. Whereas Gads is clearly required for FcεRI-mediated allergic responses, its role in T cell-mediated immunity is complex, and not easily characterized. This difficulty arises from the marked developmental defects of Gads-deficient thymocytes, which confound the interpretation of its function in peripheral T cells. Mice bearing an inducible-deletion of Gads, currently under development in our lab, will be a valuable tool for resolving this issue. A second difficulty concerns the multiple regulatory pathways intersecting at Gads. Gads-deficient mice lack both its positive and negative regulatory inputs, which may partly explain their mild phenotype. Third, we must consider the evidence that some functions of LAT and SLP-76 appear to be Gads-independent, whereas some functions of Gads may occur outside of the LAT-nucleated complex. It remains to be seen whether the Gads-like phenotypes exhibited by some mouse strains can be seen as defining a new regulatory pathway, dependent on Gads, Itk and possibly Shc, or whether their shared phenotypes merely reflect the expected outcome of partial impairment of TCR signaling. Finally, it is intriguing to consider the N-terminal SH3 of Gads, a domain that plays no role in bridging SLP-76 to LAT, and has no identified binding partners or biological functions, but is nevertheless strongly conserved throughout evolution. The study of this region may uncover new signaling functions that will help to illuminate the puzzle of Gads.

## Author Contributions

DY researched and wrote this article and designed all of the figures therein.

### Conflict of Interest Statement

The author declares that the research was conducted in the absence of any commercial or financial relationships that could be construed as a potential conflict of interest.
