# Supplementary material for: Bridging the Gap: Modulatory Roles of the Grb2-Family Adaptor, Gads, in Cellular and Allergic Immune Responses
Source: Front Immunol. 2019 Jul 25;10:1704. doi: 10.3389/fimmu.2019.01704 (PMC6669380; doi:10.3389/fimmu.2019.01704)
Supplement: Supplementary file 1 [file Table_1.docx]

**
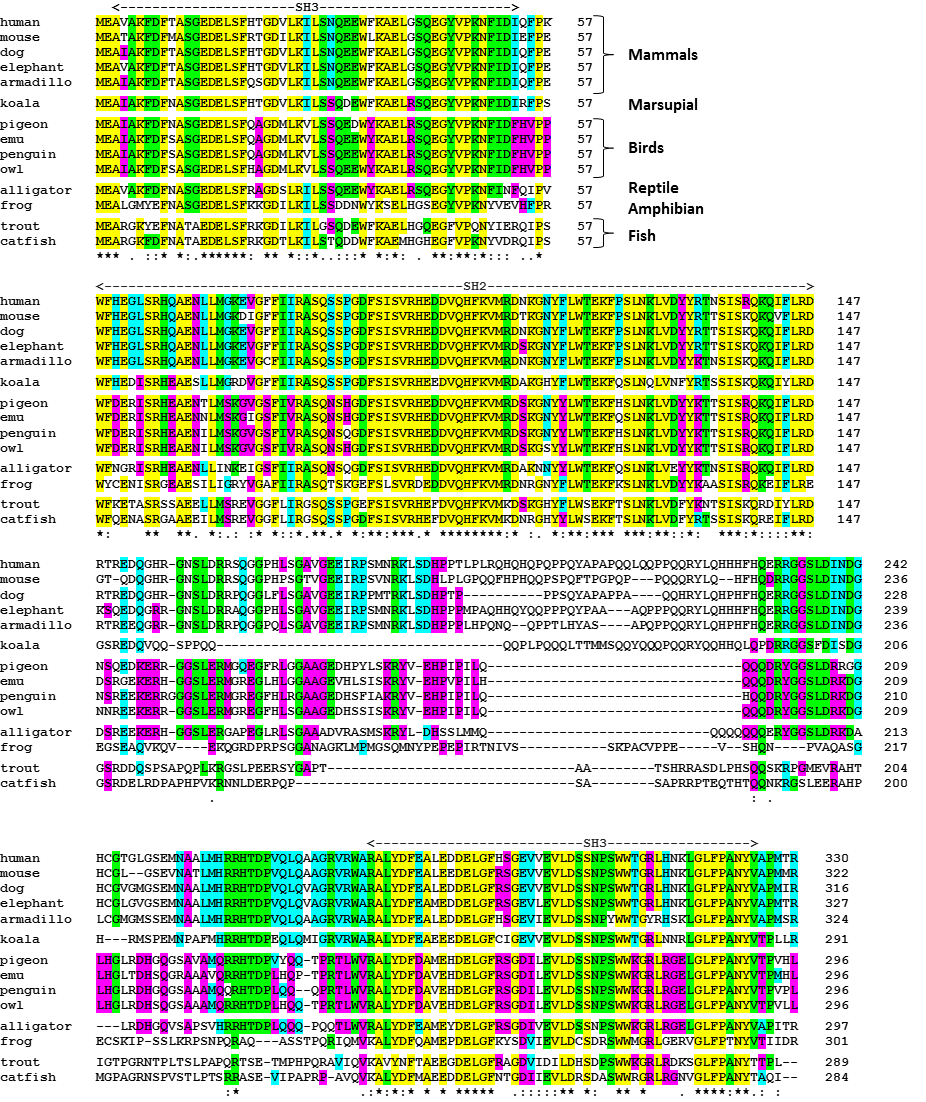
**

**Supplementary Figure 1.** **Alignment of representative Gads orthologs.** Clustal Omega was used to align the 66 vertebrate Gads orthologs listed in Table 1. Shown is an alignment of 14 representative species encompassing a wide spectrum of vertebrate diversity. Color coding is based on the conservation of residues over all 66 species (yellow, identical in at least 63 of 66 orthologs; green, identical in at least 47 of 50 avian and mammalian orthologs (excluding marsupials); blue, identical in at least 25 of 27 mammalian orthologs (excluding marsupials); pink, identical in at least 21 of 23 avian orthologs).
