# Supplementary material for: Bridging the Gap: Modulatory Roles of the Grb2-Family Adaptor, Gads, in Cellular and Allergic Immune Responses
Source: Front Immunol. 2019 Jul 25;10:1704. doi: 10.3389/fimmu.2019.01704 (PMC6669380; doi:10.3389/fimmu.2019.01704)
Supplement: Supplementary file 2 [file Table_2.docx]

| **Vertebrate Classification** |  | **Common Name** | **Latin Name** | **Order** | **Accession number** |
| --- | --- | --- | --- | --- | --- |
| **Mammalian** | 1 | Cape golden mole | Chrysochloris asiatica | Afrosoricida | XP_006865321 |
|  | 2 | Cow | Bos taurus | Artiodactyla | NP_001179489 |
|  | 3 | Pig | Sus scrofa |  | XP_013852959 |
|  | 4 | **Dog** | **Canis lupus familiaris** | **Carnivora** | **XP_849706** |
|  | 5 | California sea lion | Zalophus californianus |  | XP_027449274 |
|  | 6 | Polar bear | Ursus maritimus |  | XP_008703755 |
|  | 7 | Amur tiger | Panthera tigris altaica |  | XP_007079484 |
|  | 8 | Yangtze finless porpoise | Neophocaena asiaeorientalis | Cetacea | XP_024593314 |
|  | 9 | Yangtze River dolphin | Lipotes vexillifer |  | XP_007454055 |
|  | 10 | Sperm whale | Physeter catodon |  | XP_007102392 |
|  | 11 | Big brown bat | Eptesicus fuscus | Chiroptera | XP_008143127 |
|  | 12 | **Nine-banded armadillo** | **Dasypus novemcinctus** | **Cingulata** | **XP_004447888** |
|  | 13 | Sunda flying lemur | Galeopterus variegatus | Dermoptera | XP_008572401 |
|  | 14 | Western European hedgehog | Erinaceus europaeus | Eulipotyphla | XP_007519355 |
|  | 15 | American pika | Ochotona princeps | Lagomorpha | XP_004589543 |
|  | 16 | Cape elephant shrew | Elephantulus edwardii | Macroscelidea | XP_006890163 |
|  | 17 | Platypus | Ornithorhynchus anatinus | Monotremata | XP_001507566 |
|  | 18 | Horse | Equus caballus | Perissodactyla | XP_001502077 |
|  | 19 | Southern white rhinoceros | Ceratotherium simum simum |  | XP_004437938 |
|  | 20 | **Human** | **Homo sapiens** | **Primates** | **CAG46647** |
|  | 21 | **African savanna elephant** | **Loxodonta africana** | **Proboscidea** | **XP_003419798** |
|  | 22 | American beaver | Castor canadensis | **Rodentia** | XP_020029664 |
|  | 23 | Domestic guinea pig | Cavia porcellus |  | XP_003470559 |
|  | 24 | **House mouse** | **Mus musculus** |  | **NP_034945** |
|  | 25 | Thirteen-lined ground squirrel | Ictidomys tridecemlineatus |  | XP_005322288 |
|  | 26 | Florida manatee | Trichechus manatus latirostris | Sirenia | XP_004373937 |
|  | 27 | Aardvark | Orycteropus afer afer | Tubulidentata | XP_007939881 |
| **Marsupial** | 28 | Tasmanian devil | Sarcophilus harrisii | Dasyuromorphia | XP_003771042 |
|  | 29 | Gray short-tailed opossum | Monodelphis domestica | Didelphimorphia | XP_007503220 |
|  | 30 | **Koala** | **Phascolarctos cinereus** | **Diprotodontia** | **XP_020838328** |
| **Avian** | 31 | Chimney swift | Chaetura pelagica | Apodiformes | XP_010006109 |
|  | 32 | Okarito brown kiwi | Apteryx rowi | Apterygiformes | XP_025920792 |
|  | 33 | Chuck-will's-widow | Antrostomus carolinensis | Caprimulgiformes | XP_010168032 |
|  | 34 | Red-legged seriema | Cariama cristata | Cariamiformes | XP_009703140 |
|  | 35 | **Emu** | **Dromaius novaehollandiae** | **Casuariiformes** | **XP_025956416** |
|  | 36 | Ruff | Calidris pugnax | Charadriiformes | XP_014818956 |
|  | 37 | Speckled mousebird | Colius striatus | Coliiformes | XP_010206595 |
|  | 38 | **Rock pigeon** | **Columba livia** | **Columbiformes** | **XP_005510139** |
|  | 39 | Cuckoo roller | Leptosomus discolor | Coraciiformes | XP_009951204 |
|  | 40 | Common cuckoo | Cuculus canorus | Cuculiformes | XP_009554936 |
|  | 41 | White-tailed eagle | Haliaeetus albicilla | Falconiformes | XP_009924245 |
|  | 42 | Chicken | Gallus gallus | Galloanserae | XP_001234082 |
|  | 43 | East African grey crowned-crane | Balearica regulorum gibbericeps | Gruiformes | XP_010308889 |
|  | 44 | Red-crested turaco | Tauraco erythrolophus | Musophagiformes | XP_009982806 |
|  | 45 | White-ruffed manakin | Corapipo altera | Passeriformes | XP_027524294 |
|  | 46 | Crested ibis | Nipponia nippon | Pelecaniformes | XP_009473572 |
|  | 47 | Downy woodpecker | Picoides pubescens | Piciformes | XP_009900776 |
|  | 48 | Northern fulmar | Fulmarus glacialis | Procellariiformes | XP_009572328 |
|  | 49 | **Adelie penguin** | **Pygoscelis adeliae** | **Sphenisciformes** | **XP_009320854** |
|  | 50 | **Barn owl** | **Tyto alba** | **Strigiformes** | **XP_009972171** |
|  | 51 | White-throated tinamou | Tinamus guttatus | Tinamiformes | XP_010219648 |
|  | 52 | Anna's hummingbird | Calypte anna | Trochiliformes | XP_008494480 |
|  | 53 | Bar-tailed trogon | Apaloderma vittatum | Trogoniformes | XP_009866715 |
| **Cartaliginous Fish** | 54 | Elephant shark | Callorhinchus milii | Chimaeriformes | XP_007907975 |
| **Bony Fish** | 55 | Zebrafish | Danio rerio | Cypriniformes | NP_001017756 |
|  | 56 | Electric eel | Electrophorus electricus | Gymnotiformes | XP_026858616 |
|  | 57 | **Striped catfish** | **Pangasianodon hypophthalmus** | **Siluriformes** | **XP_026765658** |
|  | 58 | Asian bonytongue | Scleropages formosus | Osteoglossiformes | KPP67820 |
|  | 59 | Northern pike | Esox lucius | Esociformes | XP_010890046 |
|  | 60 | Atlantic salmon | Salmo salar | **Salmoniformes** | NP_001133907 |
|  | 67 | **Rainbow trout** | **Oncorhynchus mykiss** |  | **XP_021481166** |
|  | 62 | Spotted gar | Lepisosteus oculatus | Semionotiformes | XP_006636923 |
| **Amphibian** | 63 | **African clawed frog** | **Xenopus laevis** | **Anura** | **XP_018114331** |
| **Reptile** | 64 | **American alligator** | **Alligator mississippiensis** | **Crocodylia** | **XP_006264088** |
|  | 65 | Gekko | Gekko japonicus | Squamata | XP_015274651 |
|  | 66 | Western painted turtle | Chrysemys picta bellii | Testudines | XP_023955827 |

**Supplementary Table 1.** **Vertebrate Gads (Grap2) orthologs.** The NCBI Protein Blast program was used to identify and select 66 Gads orthologs from the mammalian, avian, cartilaginous and bony fish, amphibian, and reptilian classes, including representatives of 55 different taxonomical orders The 14 species listed in bold were included in the representative alignment, shown in Supplementary Figure 1.
